# SABCS 2016: systemic therapy for metastatic breast cancer

**DOI:** 10.1007/s12254-017-0326-4

**Published:** 2017-04-04

**Authors:** Simon Peter Gampenrieder, Gabriel Rinnerthaler, Richard Greil

**Affiliations:** 1 IIIrd Medical Department, Paracelsus Medical University Salzburg, Müllner Hauptstraße 48, 5020 Salzburg, Austria; 2 Salzburg Cancer Research Institute with Laboratory of Immunological and Molecular Cancer Research and Center for Clinical Cancer and Immunology Trials, Salzburg, Austria; 3 Cancer Cluster Salzburg, Salzburg, Austria

**Keywords:** Metastatic breast cancer, San Antonio Breast Cancer Symposium, SABCS, Highlights, Review

## Abstract

At the 2016 San Antonio Breast Cancer Symposium, several interesting phase II and phase III studies investigating systemic therapies for metastatic breast cancer were presented. The PrEGOC 0102 trial demonstrated that the combination of fulvestrant plus everolimus is safe and effective and could be an alternative to exemestane plus everolimus for selected patients with hormone-receptor positive, HER2-negative disease. The pan-PI3K inhibitor buparlisib showed some activity in combination with fulvestrant after failure of everolimus in the BELLE-3 trial. PIK3CA mutation detected in circulating tumor DNA (ctDNA) was predictive for a buparlisib efficacy. Unfortunately, the unfavorable toxicity profile precludes further development of this drug. Nonetheless, PI3K seems to be a valid target in tumors resistant to mTOR inhibition. The BROCADE phase II trial failed to show a statistically significant benefit by the addition of the PARP inhibitor veliparib to carboplatin and paclitaxel in patients with BRCA1/2 germline mutation. The overall response rate, however, was statistically significant higher in the veliparib arm compared to the placebo arm. Data from the phase III trial BROCADE-3 are awaited. Finally, the TNT trial did not identify further biomarkers, in addition to BRCA1/2 germline mutation, for carboplatin benefit in patients with metastatic triple-negative breast cancer.

## Introduction

Since metastatic breast cancer is generally a systemic and incurable disease, cancer drugs like endocrine agents, chemotherapy, monoclonal antibodies, and small molecules given sequentially as mono or combination treatments represent the mainstay of therapy for this disease. At the 2016 San Antonio Breast Cancer Symposium (SABCS) several trials which were designed to overcome drug resistance or to target specific vulnerabilities of breast cancer like BRCA mutation were presented. The split-up of breast cancer into distinct subgroups prevents a “one fits all” therapy but requires trial designs addressing specific questions in defined subgroups. Only a differentiated reflection of all these data allows the transmission of such trial results into daily practice.

## PrECOG 0102 phase II trial: fulvestrant plus everolimus (mTORi) or placebo after failure of an aromatase inhibitor

Postmenopausal women with hormone-receptor positive, HER2-negative locally advanced or metastatic breast cancer, resistant to an aromatase inhibitor (AI) were included in this phase II trial [1]. Patients (*n* = 129) were randomized to fulvestrant plus everolimus or fulvestrant plus placebo at standard doses. After an induction phase of 48 weeks, patients were unblinded and those without tumor progression or unacceptable toxicity continued therapy in a continuation phase. As expected, the everolimus-containing arm had a higher rate of adverse events; however no new safety signals for the mTOR inhibitor were seen. The most common side effects associated with everolimus were stomatitis (52% of patients, 9% grade 3), fatigue (42%, 6% grade 3), anemia (28%, 3% grade 3), hyperglycemia (21%, 5% grade 3), and hypertriglyceridemia (32%, 3% grade 3). The everolimus-containing arm showed a significantly longer progression-free survival (PFS) compared fulvestrant alone (median PFS 10.4 vs. 5.1 months; hazard ratio [HR] 0.60, 95%CI 0.40–0.92; *P* = 0.02). There was no difference in overall survival (HR 1.11); however, follow-up was short and the trial was not powered to detect a survival difference. In conclusion, fulvestrant plus everolimus is an effective combination with a moderate toxicity profile and it could be an alternative to exemestane plus everolimus for selected AI-resistant patients. The label for this combination, however, is not available yet.

## BELLE-3 phase III trial: fulvestrant plus buparlisib (pan PI3KI) or placebo after failure of everolimus

Following inhibition of the mammalian target of rapamycin complex 1 (mTORC1), the main target of everolimus, feedback-loops can reactivate phosphoinositide 3‑kinase (PI3K) which lies upstream to mTOR in the PI3K/AKT/mTOR pathway [2]. Therefore, inhibition of this protein seems reasonable after failure of everolimus. The BELLE‑3 trial investigated the pan-PI3K inhibitor buparlisib (BKM120) in combination with fulvestrant in comparison to fulvestrant plus placebo. Postmenopausal women with hormone-receptor positive, HER2-negative locally advanced or metastatic breast cancer who progressed on or after a mTOR inhibitor as last line of treatment were included (*n* = 432). Two thirds of the patients had visceral metastases and were pretreated with at least two lines of endocrine therapy for metastatic disease. Median duration of prior mTOR inhibition (99% everolimus, 1% ridaforolimus) was 8.0 months in the experimental arm and 8.6 months in the control arm. In the buparlisib-containing arm, the median PFS was statistically significant prolonged (3.9 vs. 1.8 months; HR 0.67; 95%CI 0.53–0.84; *P* < 0.001). The overall response rate was 7.6% with the PI3K inhibitor and 2.1% with fulvestrant alone. Unfortunately, buparlisib had an unfavorable toxicity profile with hyperglycemia, nausea, diarrhea, fatigue, and elevated liver enzymes, but also psychiatric side effects like depression and anxiety. Three cases of suicide attempts were reported in the experimental arm vs. none in the placebo plus fulvestrant arm. In the subgroup analyses, only patients with visceral disease benefited from the PI3K inhibition; however such analyses must be interpreted with caution. Promising results came from the biomarker research program: a mutation in the gene coding for PI3K (PIK3CA) was predictive for a longer PFS by buparlisib. This effect was seen for mutations detected in the primary tumor (HR 0.39; 95%CI 0.23–0.65; *P* < 0.01) as well as in the circulating tumor DNA (ctDNA; HR 0.46; 95%CI 0.29–0.73; *P* < 0.01). A similar effect was already seen in the BELLE-2 trial investigating buparlisib plus fulvestrant after failure of an aromatase inhibitor. The BELLE‑3 study demonstrated the proof-of-principle of PI3K inhibition after everolimus failure and hopefully the currently investigated alpha-specific PI3K inhibitors have a more favorable therapeutic index.

## BROCADE phase II trial: paclitaxel/carboplatin plus veliparib (PARPi) or placebo in patients with MBC and BRCA1 or BRCA2 mutations

A mutation in BRCA1 or BRCA2 leads to a deficiency in the homologous recombination (HR) repair pathway for DNA double-strand breaks [3]. Inhibition of PARP (poly ADP ribose polymerase) blocks alternative mechanisms of DNA repair leading to multiple double-strand breaks and cell death. Veliparib, a selective high potent PARP inhibitor, was tested in the randomized phase BROCADE II trial in combination with paclitaxel and carboplatin vs. the same chemotherapy regimen plus placebo. Patients with locally recurrent or metastatic breast cancer harboring a BRCA1/2 mutation were included (*n* = 196). Most of the patients had HER2-negative (95%) and 82 (41%) had triple-negative breast cancer. The main toxicities were hematologic; however, no meaningful differences were detected between the two treatment arms neither for hematologic or for nonhematologic toxicities. Given the favorable toxicity profile of veliparib, the rate of dose interruptions, dose reductions, and treatment discontinuations due to adverse events were similar between the two arms. The primary endpoint, progression-free survival (PFS), however, was not statistically different between the veliparib and the placebo arms (14.1 vs. 12.3 months, HR 0.79, 95%CI 0.54–1.16; *P* = 0.231). Furthermore, there was no difference in overall survival, but the data were still immature. The overall response rate, in contrast, was statistically significant higher in the veliparib arm compared to the placebo arm (78% vs. 61%; *P* = 0.027). A randomized phase III trial (BROCADE‑3) is ongoing investigating veliparib in combination with weekly paclitaxel and carboplatin (NCT021163694).

## PERTAIN phase II trial: an aromatase inhibitor plus trastuzumab ± pertuzumab in first-line patients with HER2-positive and hormone receptor-positive MBC

The crosstalk between estrogen receptor signaling and HER2 signaling is thought to be an escape mechanism of breast cancer cells under the pressure of anti-HER2 therapy or endocrine therapy, respectively [4]. Therefore the combination of an aromatase inhibitor with anti-HER2 therapy is reasonable and was investigated in this randomized phase II trial. Postmenopausal patients with HER2-positive, ER-positive metastatic breast cancer were randomly assigned to an aromatase inhibitor plus trastuzumab with or without pertuzumab (*n* = 258). At the discretion of the investigator, patients could receive a chemotherapy induction phase with a taxane plus trastuzumab ± pertuzumab prior to the start of endocrine therapy. This was applied to 56% of patients. In the intention-to-treat population, the addition of pertuzumab led to a statistically significant increase of median PFS from 15.8 months to 18.9 months (HR 0.65, 95%CI 0.48–0.89; *P* = 0.007). Subgroup analyses were generally consistent with the primary analysis. Pertuzumab plus trastuzumab plus an aromatase inhibitor was well tolerated and no new safety signals were reported, making this combination a valid treatment option for selected patients. For patients without contraindication for chemotherapy, induction chemotherapy in combination with trastuzumab and pertuzumab remains the standard of care. If the addition of endocrine therapy to dual HER2-blockade further improves efficacy remains unclear, since this question was not addressed in the PERTAIN trial.

## TNT phase III trial: carboplatin vs. docetaxel in TNBC – secondary endpoints

The TNT trial randomized 376 patients with triple-negative metastatic or locally advanced breast cancer to 6 cycles of docetaxel 100 mg/m^2^ or carboplatin AUC 6 [5]. In the overall population as well as in the subgroup of patients without BRCA1/2 germline mutation, no significant difference in terms of overall response rate (ORR) was seen between the two treatment arms (31.4% for carboplatin, 35.6% for docetaxel; *P* = 0.44). For patients harboring a BRCA1 or 2 germline mutation, however, carboplatin led to a statistically significant higher response rate compared to docetaxel (68.0% vs. 33.3%; *P* = 0.03). These data were already presented two years ago [6]. At the SABCS 2016, further potential biomarkers for carboplatin efficacy were presented: BRCA1 gene methylation, BRCA1 silencing measured at the mRNA level and high HRD (homologous recombination deficiency) score. Unexpectedly, none of these markers had any predictive value for carboplatin response. For all three patient groups, docetaxel showed a numerically higher ORR (42.1% vs. 21.4% for BRCA1 methylation, 64.7% vs. 28.6% for BRCA1 silencing and 42.6% vs. 38.2% for high HRD score). The explanation for this phenomenon remains speculative. Since all biomarkers were assessed in the primary tumor, the authors supposed that unlike germ-line mutation, BRCA1 methylation is changeable over time and could be lost during adjuvant therapy or the metastatic process.

## Conclusion

None of the presented trials will immediately change clinical practice, but all these data represent further pieces in the jigsaw puzzle of metastatic breast cancer.

## Abbreviations

AUC: Area under the curve
BRCA1/2: Breast cancer gene 1 and 2
ctDNA: Circulating tumor DNA
DNA: Deoxyribonucleic acid
HER2: Human epidermal growth factor receptor 2
HR: Hazard ratio
HRD: Homologous recombination deficiency
MBC: Metastatic breast cancer
mRNA: Messenger ribonucleic acid
mTOR: Mechanistic (or mammalian) target of rapamycin
mTORC1: Mammalian target of rapamycin complex 1
ORR: Overall response rate
PARP: Poly (ADP-ribose) polymerase
PFS: Progression-free survival
PI3K: Phosphoinositide-3-kinase
PIK3CA: Phosphatidylinositol 3‑kinase catalytic 110-KD alpha
SABCS: San Antonio Breast Cancer Symposium
TNBC: Triple-negative breast cancer
